# Supplementary material for: Individualized Goal Setting for Pediatric Intensive Care Unit-Based Rehabilitation Using the Canadian Occupational Performance Measure
Source: Children (Basel). 2023 May 31;10(6):985. doi: 10.3390/children10060985 (PMC10297534; doi:10.3390/children10060985)
Supplement: Supplementary file 1 [file children-10-00985-s001.zip › children-2268777-supplementary.pdf]

Supplementary Material

Article

Individualized Goal Setting for Pediatric Intensive Care Unit-based Rehabilitation Using the Canadian Occupational Performance Measure

Youngsub Hwang [1], Jeong-Yi Kwon [2*], Joongbum Cho [3] and Jaeyoung Choi [3]

1 Department of Health Sciences and Technology, Samsung Advanced Institute for Health Sciences and Technology, Sungkyunkwan University, Seoul, Republic of Korea
2 Department of Physical and Rehabilitation Medicine, Sungkyunkwan University School of Medicine, Samsung Medical Center, Seoul, Republic of Korea
3 Department of Critical Care Medicine, Sungkyunkwan University School of Medicine, Samsung Medical Center, Seoul, Republic of Korea
* Correspondence: jeongyi.kwon@samsung.com; Tel.: +82-4591-1039

Table of contents

Table S1. Summary of responses to COPM interview that generated as functional goals

Table S2. Summary of responses to COPM interview that generated as personal factors

Table S3. Clinical indicators for high number of problem lists generated as occupational performance prediction equation in pediatric intensive care units

Table S4. Comparison of sensitivity and specificity between the training dataset and test dataset with the developed prediction equation program

Table S1. Summary of responses to COPM interview that generated as functional goals

Goal domains	Examples of caregiver's answer
**Functional mobility**	
Actively repositioning in bed	"I wish my child could perform side-lying positioning with a maximal assistance to prevent bedsores."
Sitting whilst leaning back	"I hope my child to be able to sit in bed with leaning his back by himself, because then he will be able to engage more activities."
Sitting on the edge of the bed	"I think my child needs to perform sitting on edge of bed himself to engage in sit-to-stand training session."
Transfer	"I hope my child could participate bed to wheelchair transfer with a maximal assistance."
Reaching	"I wish she could reach out and grab any objects that are within her reach."
Ambulation	"I wish my child could ambulate using a walker with maximal assistance from more than two people."
**Personal care**	
Communication	"I wish my child could express his intentions with hand signals or head shakes because I want to know my child's needs."
Personal hygiene	"I wish my child could participate in bed-bath to prevent bedsores and infections."
Dressing	"I wish my child could wear a mask himself to prevent virus or any infections."
**Quiet recreation**	
Using electronic devices	"I wish my child could use cellphone so he can play some games for leisure and communicate with us more frequently."

Table S2. Summary of responses to COPM interview that generated as personal factors

Goal domains	Examples of caregiver's answer
**Physiological factor**	
Respiratory function	"I wish my child could breathe herself without a ventilator."
Consciousness	"I hope my child recovers to a level of consciousness that he can respond to being pinched."
Intubation, IV nutrition	"I hope my child recovers to the point intubation can be removed, because it seems so uncomfortable for him."
Urinary catheterization	"I hope my child recovers to the point that urinary catheterization can be removed. It's confusing because so many lines are connected to him."
Sensory	"I hope my child's delirium symptom get better."
Body temperature	"I wish his body temperature to go down."
Cough	"I hope my child stops coughing."
Swelling	"I hope the swelling on his both legs reduces."
**Physical factor**	
Muscle strength	"I wish my child to recover more than 50% of her strength before admission to PICU."
Stiffness	"I wish the stiffness in both his legs reduces."
Range of motion	"I wish my child to recover more than 50% of her range of motion of both legs and arms before admission to PICU."

Table S3. Clinical indicator for high number of problem lists generated as occupational performance prediction equation in pediatric intensive care units

Target	Var	Beta	Standard error	P-value
nOP ≥4	Intercept	5.2961	3.4267	0.122
	STAI-X-1	-0.1429	0.0699	0.041

Stepwise variable selection method in logistic regression ($P < 0.05$). nOP, number of problem lists generated as occupational performance; STAI-X-1, State-Trait Anxiety Inventory

Table S4. Comparison of sensitivity and specificity between the training dataset and test dataset with the developed prediction equation program

Target	Cutoff	TP	FN	FP	TN	AUC	95% CI of the AUC		Sensitivity	Specificity	J
nOP ≥4	0.08	4	0	9	28	0.878	0.808	0.948	1.000	0.757	0.757

TP, true positives; FN, false negatives; FP, false positives; TN, true negatives; J, Youden index; CI, confidence interval; AUC, area under the curve; nOP, number of problem lists generated as occupational performance
